# Construction of *E*. *coli—Mycobacterium* shuttle vectors with a variety of expression systems and polypeptide tags for gene expression in mycobacteria

**DOI:** 10.1371/journal.pone.0230282

**Published:** 2020-03-11

**Authors:** Surya Pratap Seniya, Priya Yadav, Vikas Jain

**Affiliations:** Microbiology and Molecular Biology Laboratory, Department of Biological Sciences, Indian Institute of Science Education and Research (IISER), Bhopal, India; Bose Institute, INDIA

## Abstract

Cloning and expression of a desired gene is indispensable in molecular biology studies. Expression vectors, in this regard, should offer much needed flexibility and choice of cloning strategies for both *in vivo* and *in vitro* protein expression experiments. Furthermore, availability of option to choose from various reporter tags allows one to be flexible during designing of an experiment in a more relevant manner. Thus, the need of a versatile expression system cannot be ignored. Although several different expression vectors are available for gene expression in mycobacteria, they lack the required versatility of expression and the inclusion of reporter tags. We here present the construction of a set of nine *E*. *coli*-*Mycobacterium* shuttle plasmids, which offer a combination of three mycobacterial promoter systems (heat shock inducible-*hsp60*, tetracycline-, and acetamide-inducible) along with three polypeptide tags (Green Fluorescent Protein (GFP), Glutathione S-transferase (GST) and hexa-histidine tag). These vectors offer the cloning of a target gene in all the nine given vectors in parallel, thus allowing the generation of recombinant plasmids that will express the target gene from different promoters with different tags. Here, while the hexa-histidine and GST tags can be used for protein purification and pull-down experiments, the GFP-tag can be used for protein localization within the cell. Additionally, the vectors also offer the choice of positioning of the reporter tag either at the N-terminus or at the C-terminus of the expressed protein, which is achieved by cloning of the gene at any of the two blunt-end restriction enzyme sites available in the vector. We believe that these plasmids will be extremely useful in the gene expression studies in mycobacteria by offering the choices of promoters and reporters. Our work also paves the way to developing more such plasmids with other tags and promoters that may find use in mycobacterial biology.

## Introduction

Tuberculosis (TB) is considered as one of the top 10 causes of human death. Worldwide about 1.7 billion people are infected with latent TB and 1.3 million deaths are caused by it every year. Even after 2 decades of complete genome sequencing of *Mycobacterium tuberculosis* [1] the causative agent of TB, there is no effective vaccine available against TB and most of the drug therapies are expensive and time taking. Furthermore, the emergence of drug resistant strains of *M*. *tuberculosis* makes the situation even more dreadful. Therefore, there is an urgent need to explore newer drug targets and better remedies to treat TB.

Controlled and constitutive gene expression tools provide powerful platform to identify, study, and validate drug targets in bacteria including *M*. *tuberculosis*. Although *E*. *coli* has been the most sought after bacterial system for protein expression, a large number of mycobacterial proteins do not express well in *E*. *coli*, and many a times, form inclusion bodies [2, 3]. In this regard, availability of a better and versatile mycobacterial expression system that allows for the expression of mycobacterial genes within mycobacteria in a controlled and study-demanding manner would greatly help in identifying new drug targets. Constitutive expression systems such as *Ag85* [4] and *rel* [5] are convenient to use since they do not require any inducer to initiate the expression of the target gene. However, these systems become highly undesirable when the aim is to express a lethal gene in bacteria, since the constitutive expression of the lethal gene would be detrimental for the bacteria. On the other hand, inducible promoters are very important for the controlled expression of genes. Since the development of first mycobacterial heat-inducible expression system involving *hsp60* promoter [6], many more expression systems have been developed that allow the induction of gene expression by the addition of either acetamide [7], tetracycline [8], Pristinamycin [9], or nitrile compounds [10]. In addition, a Gateway cloning vector, pDESTsmg, for gene expression in mycobacteria has been developed [11].

Most of the available mycobacterial plasmids lack generality in cloning of the desired gene with, additionally, an option of a suitable polypeptide tag at one of the ends of the expressed protein, which is crucial for monitoring the expression and localization of the target molecule *in vivo*. In this study, we have designed nine *Escherichia coli*–mycobacteria shuttle plasmids. These vectors have been developed by using pMV261 vector backbone and replacing its *hsp60* promoter with either acetamide- or tetracycline-inducible promoter. Additionally, in all of the vectors, we have installed three different reporter tags (such as 6xHis, GFP, and GST) and two restriction enzyme sites, which allow direct cloning of the blunt-end target DNA either before or ahead of the tag. These vectors offer inducible expression of the target gene with a choice of hexa-histidine, GFP, FLAG or GST tags at either N or C terminal end of the protein as required.

## Materials and methods

### Bacterial strains, media, reagents, and growth conditions

*E*. *coli* strain XL1-Blue (Stratagene) was used for all cloning experiments, and was grown in LB broth (Difco) at 37°C in the presence of 50 μg/ml kanamycin (Sigma-Aldrich) with constant shaking at 200 rpm or on LB agar plates. *M*. *smegmatis* mc^2^155 was used for mycobacterial expression studies. Plasmids generated in this study (Table 1) were electroporated in *M*. *smegmatis*, and the bacteria were selected on the required antibiotic. Bacteria were grown in Middlebrook 7H9 (Difco) liquid broth supplemented with 2% glucose and 0.05% Tween 80, at 37°C with constant shaking at 200 rpm or on Middlebrook 7H9 solid media supplemented with 2% glucose with incubation at 37°C. 25 μg/ml of kanamycin was used for mycobacterial culture. DNA modifying enzymes (such as Antarctic phosphatase, T4 polynucleotide kinase, and restriction enzymes, etc.) were procured from New England Biolabs. All the enzymes were used as per manufacturer’s instructions.

**Table 1:** List of oligos used in this study. Restriction enzyme sites, wherever present, are shown in bold letters.

S. No.	Oligo name	Sequence (5’ - 3’)
1.	pMV_His_For	CACCACCACCACCACCACAGTGGTAGT**GTTAAC**TAGCGTACGATCGACTGCCAG
2.	pMV_His_Rev	GTGGTGGTGGTGGTGGTGACTACCACTGATATC**CATATG**TGGACTCCCTTTCTCTTATCGGG
3.	pMV_NdeI_For	GTGCTGTGGTTCATGCTGTTCCTG
4.	pMV_SpeI_Rev	GTGGTGGCCTAACTACGGCTACAC
5.	pMV_GFP_For	GATGAGTCCA**CATATGGATATC**AGTGGTAGTATGAGCAAGGGCGAGGAGCTGTTC
6.	pMV_GFP_Rev	GATGTACGTCA**GTTAAC**ACTACCACTCTTGTACAGCTCGTCCATGCCGAG
7.	pMV_GST_For	GATGAGTCCA**CATATGGATATC**AGTGGTAGTATGTCCCCTATACTAGGTTATTGGAAAATTAAGGG
8.	pMV_GST_Rev	GATGTACGTCA**GTTAAC**ACTACCACTATCCGATTTTGGAGGATGGTCGCCAC
9.	B-gal_for	ATGCACTGCAGTGGGGATCCC
10.	B-gal_rev	TTTTTGACACCAGACCAACTGGTAATGG
11.	pMV_Tet	GGATCCGGAGGAAAT**CATATG**AAATTTGG
12.	pMVactseq	CCGCAGTTGTTCTCGCATAC
13.	pMVfor	GCCAGCGTAAGTAGCGG
14.	pMV_Tet_Seq	GTGACTGCTCGCTACTCTCATCTG
15.	pMVrev2	TTGATGCCTGGCAGTCGATC
16.	Bgal_NdeI_for	CGACGGTTTCCACATGGGGATTGGTGG
17.	pMV_NheI_rev	CCGTTGAATATGGCTCATAACACCC

### PCR and Site Directed Mutagenesis

PCR reactions were carried out using Phusion high fidelity DNA polymerase (NEB) as per manufacturer’s instructions. Primers used in PCR and site-directed mutagenesis (SDM) experiments are listed in Table 1, and were procured from Macrogen. SDM was carried out as described previously [12, 13]. Positive clones were confirmed by sequencing at Macrogen, South Korea.

### Protein expression analysis

To determine target gene expression in *M*. *smegmatis*, the bacterial cultures carrying the desired plasmids were grown at 37°C with constant shaking at 200 rpm. Induction of gene expression was carried out with either 2% acetamide (Sigma-Aldrich), 200 ng/ml anhydrotetracycline hydrochloride (Sigma-Aldrich), or by incubating the culture at 42°C as required. The cultures were induced at OD_600_ ~0.6–0.8 for three hours. Cell were harvested, re-suspended in phosphate-buffered saline (PBS), and then lysed by sonication. The sonicated samples were centrifuged at 14000 rpm for 15 min at 4°C, and the supernatant was collected. The samples were quantified by Bradford assay (Biorad protein Assay) and equal amounts of protein samples were separated on 12% SDS polyacrylamide gel. Proteins were then transferred on a polyvinylidene difluoride (PVDF) membrane (Millipore), and Western blotting was carried out using appropriate antibodies (anti-His, anti-GFP, or anti-GST). This was followed by probing with either anti-mouse IgG DyLight 680-conjugated secondary antibody (Thermo Scientific) or anti-rabbit IgG DyLight 800 conjugated secondary antibody (Invitrogen), as required. The blots were scanned on an Odyssey infrared imaging system (LI-COR Biosciences, Lincoln, NE). 40 μl of 40 mg/ml 5-bromo-4-chloro-3-indolyl-β-D-galactopyranoside (X-gal) was used in MB7H9-agar plates for the blue white screening.

### Microscopy imaging

Fluorescence microscopy imaging was carried out on Leica DM2500 system equipped with 100X PL APO objective. FM4-64 (N-(3-Triethylammoniumpropyl)-4-(6-(4-(Diethylam- ino) Phenyl) Hexatrienyl) Pyridinium Dibromide) was obtained from Invitrogen, and was used following the supplier’s instructions. Acetamide-induced culture of *M*. *smegmatis* carrying pMA-GFP was counterstained with FM4‐64 membrane staining dye for 30 min, washed thrice with PBST (Phosphate buffered saline with Tween-20), resuspended in 0.1 ml of PBST, and imaged. The imaging was carried out by using GFP (488 nm) and FM4‐64 (506 nm) filters.

## Results

Here we present the construction of a set of nine *E*. *coli*-mycobacteria shuttle plasmids that can be used for the expression of genes in mycobacteria. They carry three different promoter systems (*hsp60*, tetracycline-, and acetamide-inducible) with three different polypeptide tags (hexa-histidine, GST, and GFP). Additionally, the genes can be cloned in this vector in such a way that the expressed protein can have the tag at its N- or C-terminus.

### Construction of acetamide-inducible plasmid with polypeptide tags

**Fig 1:**
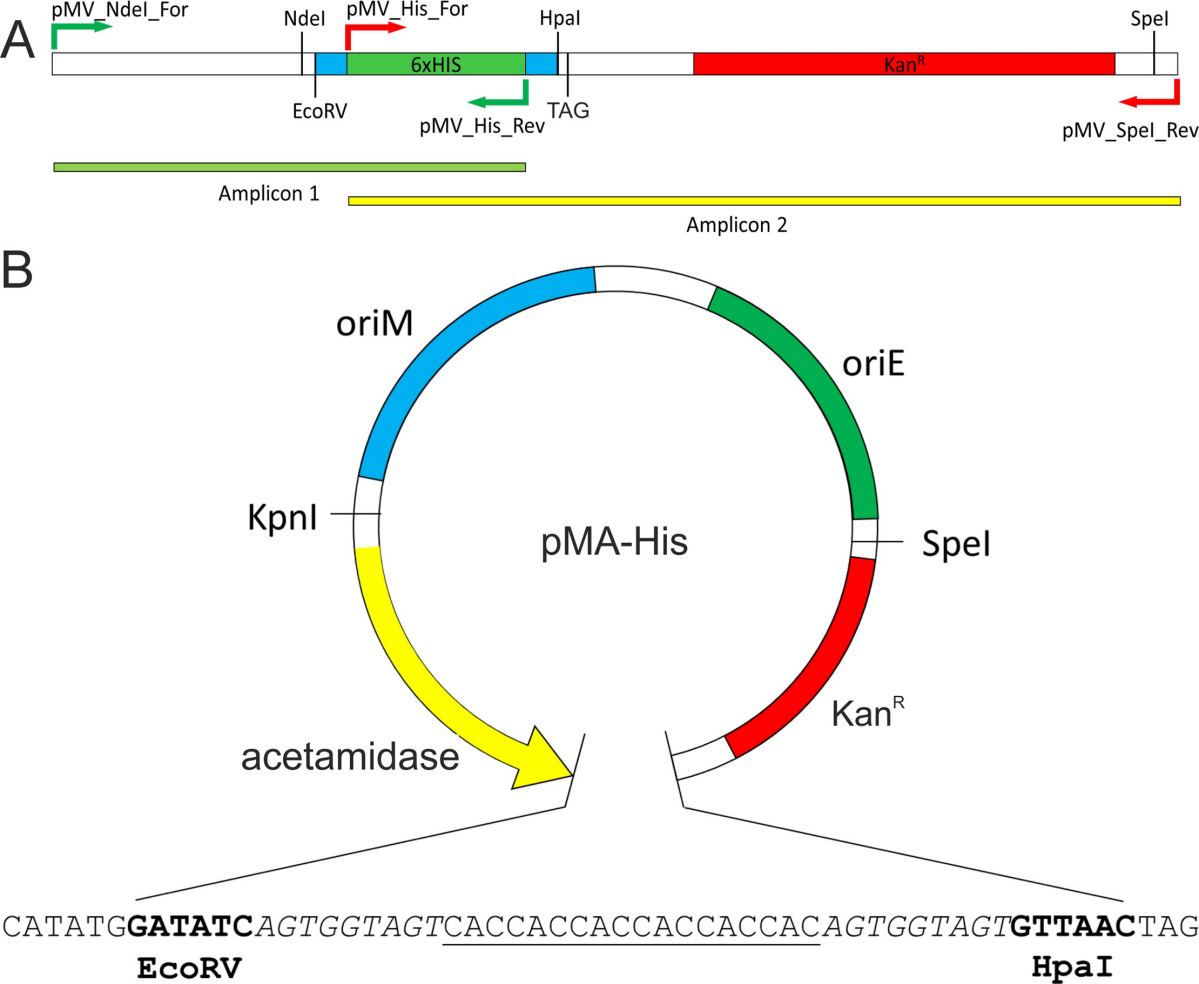
Schematic representation of proposed vector with pMA-His as example. Panel A depicts the PCR-generated amplicons of 6x-His fragment (amplicon 1; green bar) and the kanamycin resistance marker (amplicon 2; yellow bar). Primers used in these PCR reactions are shown as arrows. TAG (stop codon) and relevant restriction enzyme sites are shown. Panel B shows the graphical representation of proposed plasmid with acetamide-inducible promoter system (labelled as acetamidase). *oriM* and *oriE* represent the origin of replication for mycobacteria and *E*. *coli*, respectively. Kan^R^ is the kanamycin resistance marker. The nucleotide sequence of the region between EcoRV and HpaI is shown having the following elements: NdeI–EcoRV–Ser-Gly-Ser linker (italicized sequence)–reporter tags (either 6x-His, GFP or GST)–Ser-Gly-Ser linker (italicized sequence)–HpaI–Stop codon. For presentation purpose, 6x-histidine tag is shown as underlined sequence.

We had previously reported the construction of an acetamide-inducible plasmid, pMVAcet, which was used for the expression of various genes in *M*. *smegmatis* [14–16]. Here, we cloned various polypeptide tag-coding sequences in pMVAcet vector and further replaced the promoter with others inducible systems to prepare our plasmids (Table 2). Primers pMV_NdeI_For and pMV_His_Rev were used to amplify amplicon1 (Fig 1A), whereas amplicon 2 was amplified with primers pMV_His_For and pMV_SpeI_Rev (Fig 1A) using pMVAcet as template. An overlapping PCR was then carried out with pMV_NdeI_For and pMV_SpeI_Rev primers to join amplicon 1 and amplicon 2 to generate 6xHis tag DNA fragment with Ser-Gly-Ser linker on both sides of the 6xHis tag followed by kanamycin resistance marker (Fig 1A). The generated fragment was treated with NdeI and SpeI enzymes and ligated to pMVAcet vector backbone pre-digested with the same enzymes; 6xHis fragment was amplified with Kan^r^ for ease of cloning and screening, since vector backbone digested with NdeI and SpeI lacks kanamycin resistance marker which would be provided by insert, and, therefore, colonies will only appear in case of efficient ligation of the insert. The generated vector was named as pMA-His (Fig 1B).

**Table 2:** List of plasmids used in this study. All the plasmids carry kanamycin resistance marker. pMA, pMT, and pMH represent plasmids carrying acetamidase, *tetRO*, and *hsp60* promoter, respectively. 6xHis, hexa-histidine tag; GFP, Green Fluorescent Protein; GST, Glutathione S-transferases tag.

S. No.	Name	Backbone	promoter	Reporter tag	Source
1.	pMVAcet	pMV261	*acetamidase*	None	Lab material
2.	pMEND-Lx	pMINDLx	*tetRO*	LuxAB	Addgene
3.	pMVTet	pMV261	*tetRO*	None	This Study
4.	*pMH-His	pMV261	*hsp60*	6xHis	This Study
5.	pMH-GFP	pMV261	*hsp60*	GFP	This Study
6.	pMH-GST	pMV261	*hsp60*	GST	This Study
7.	*pMA-His	pMVAcet	*acetamidase*	6xHis	This Study
8.	pMA-GFP	pMVAcet	*acetamidase*	GFP	This Study
9.	pMA-GST	pMVAcet	*acetamidase*	GST	This Study
10	pMT-His	pMVTet	*tetRO*	6xHis	This Study
11.	pMT-GFP	pMVTet	*tetRO*	GFP	This Study
12.	pMT-GST	pMVTet	*tetRO*	GST	This Study
13.	pMA-βgal-His	pMVAcet	*acetamidase*	6xHis	This Study
14.	pMT-βgal-His	pMVTet	*tetRO*	6xHis	This Study
15.	pMH-βgal-His	pMV261	*hsp60*	6xHis	This Study
16.	pSD5b	pSD5b	NA	*lacZ*	Lab material
17.	pMS-QS-NOHGFP	pET21b	T7 promoter	GFP	Lab material

*pMH-His and pMA-His were earlier named as pSS1 and pSS4, respectively [17].

Next, to clone GFP and GST polypeptide tags in pMVAcet plasmid, GFP was amplified using pMS-QS-NOHGFP vector [12] as template with pMV_GFP_For and pMV_GFP_Rev primers. The GST fragment was amplified using pGEX-4t.2 plasmid (GE Healthcare) as template with pMV_GST_For and pMV_GST_Rev primers. The amplified GFP and GST fragments were digested with NdeI and HpaI enzymes and ligated to dephosphorylated vector backbone (prepared by digesting pMVAcet plasmid with NdeI and HpaI), giving rise to pMA-GFP and pMA-GST, respectively.

### Construction of heat- and tetracycline-inducible vectors with polypeptide tags

**Fig 2:**
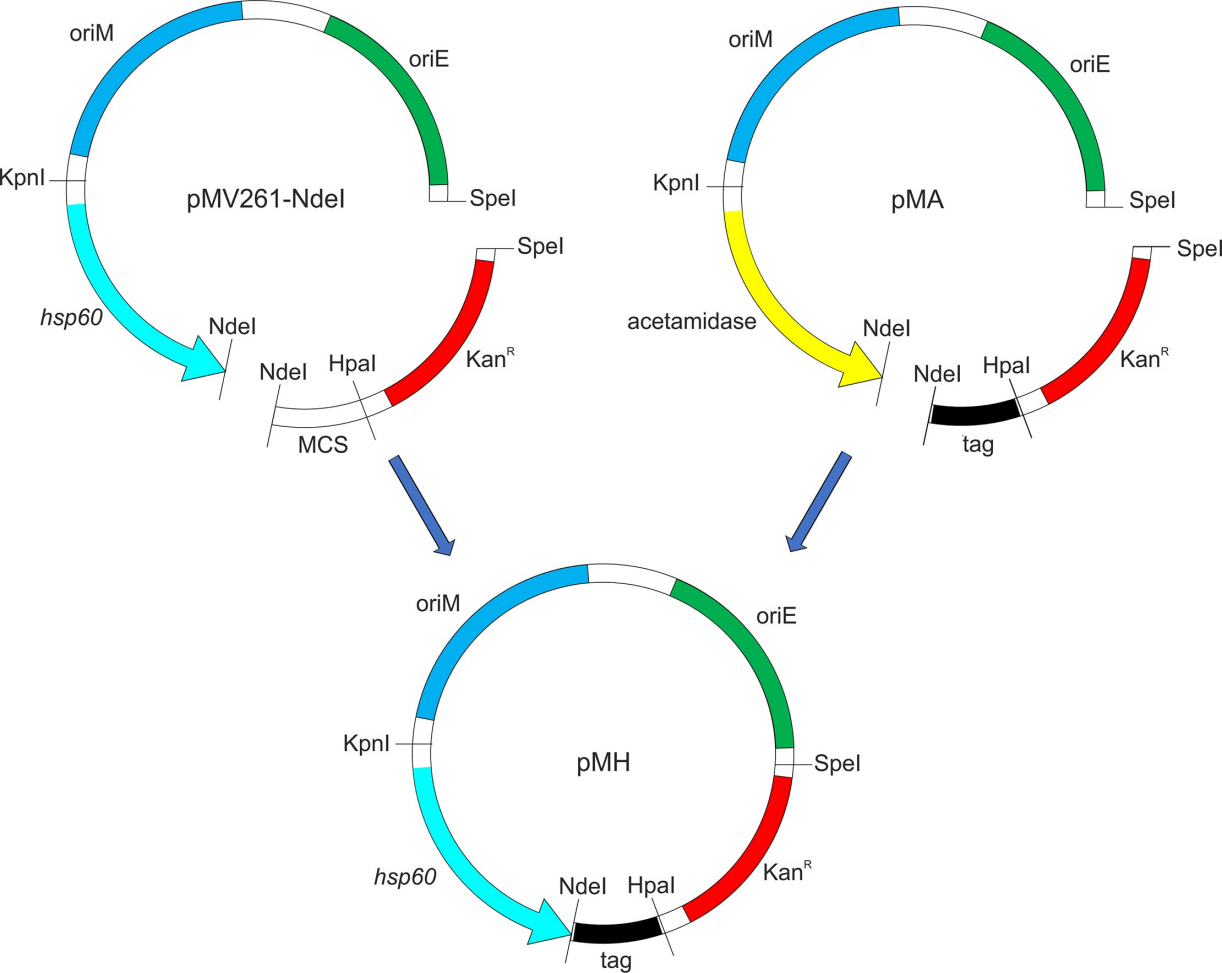
Schematic representation of subcloning of various tags from pMA tag vectors to heat inducible promoter systems. Respective tag-containing fragments (6xHis, GFP or GST) were obtained by digestion of pMA-His, pMA-GFP, or pMA-GST vectors by NdeI and SpeI (shown in the vectors) and ligated downstream of *hsp60* promoter at the same sites. *oriM* and *oriE* represent the origin of replication for mycobacteria and *E*. *coli*, respectively. Kan^R^ is the kanamycin resistance marker. Similarly, cloning of these tags was carried out under *tetRO* promoter, thus giving rise to pMT vectors.

To prepare a tetracycline-inducible plasmid in the pMV261 vector backbone with desired polypeptide tags, we used pMEND-Lx vector [18]. pMEND-Lx was a gift from Brian Robertson (Addgene plasmid #25010; http://n2t.net/addgene:25010; RRID:Addgene_25010). We derived the tetracycline-inducible promoter system (*tetRO*) from the pMEND-Lx vector. This was achieved by introducing an NdeI restriction enzyme site in pMEND-Lx vector by site-directed mutagenesis using the primer pMV_Tet. Next, the modified pMEND-Lx vector was digested with KpnI and NdeI enzymes, and the released insert carrying the *tetRO* promoter system was introduced in pMV261 vector at the same sites, thus giving rise to pMVTet plasmid (S1 Fig). It may be noted here that the pMV261 vector used here was modified in our laboratory to carry NdeI site [14]. To clone 6xHis, GFP, and GST tags under *hsp60* and tetracycline-inducible promoters, respective tags along with kanamycin resistance marker were excised from pMA-His, pMA-GFP, and pMA-GST using NdeI and SpeI enzymes, and introduced in pMV261 and pMVTet vectors, at the same sites, resulting in six different plasmids having two promoter systems with three polypeptide tags each (Fig 2).

### Functional assessment of the promoters in the generated plasmids

**Fig 3:**
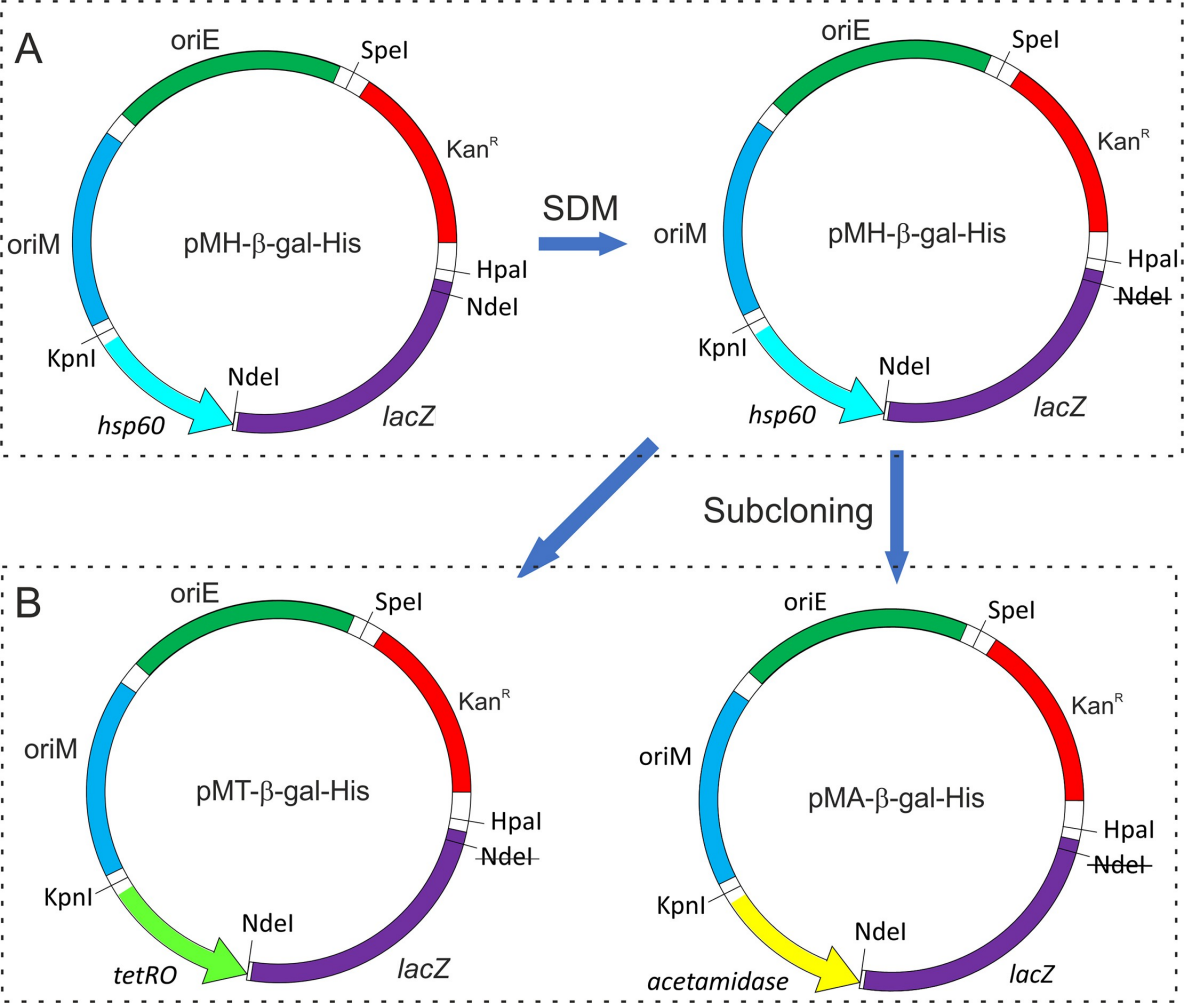
Schematic representation of cloning of *lacZ* gene in given plasmids. Panel A depicts site-directed mutagenesis to abolish NdeI restriction enzyme site (shown as strikethrough) within *lacZ* gene. Panel B depicts subcloning of *lacZ* gene in pMT-HIS and pMA-HIS vectors, which was carried out using NdeI and SpeI enzymes. Here, *tetRO* and *acetamidase* represent the tetracycline- and acetamide-inducible promoters. *oriM*, *oriE*, and Kan^R^ represent the origin of replication for mycobacteria and *E*. *coli*, and the kanamycin resistance marker, respectively.

**Fig 4:**
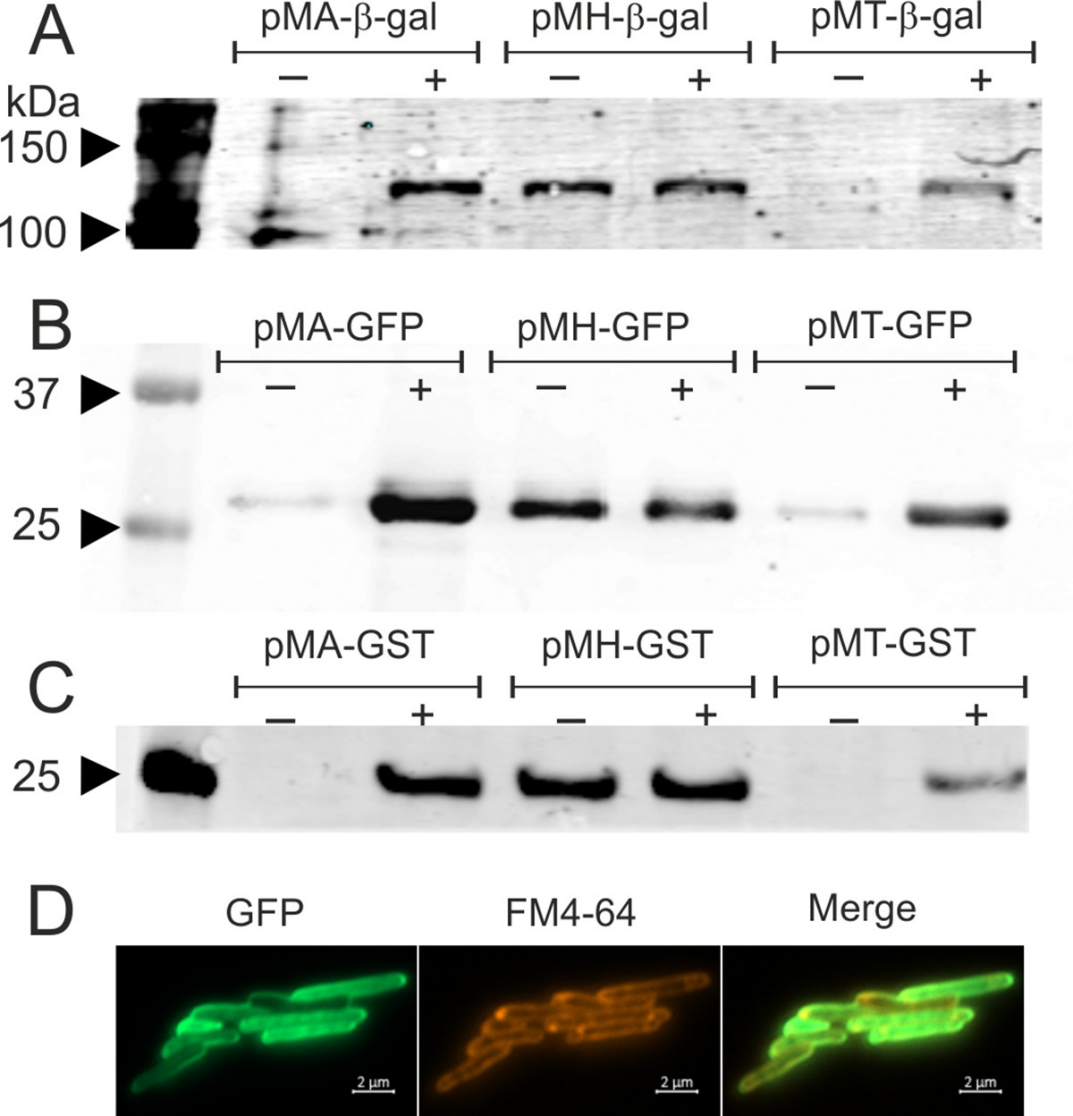
Expression of *lacZ* and other tags under different promoter systems. The Western blot data presented here depict the production of β-galactosidase (Panel A), GFP (Panel B), and GST (Panel C). Expression in each case is shown under acetamidase, *hsp60*, and *tetRO* promoters, in presence (+) and absence (-) of the inducer. In each panel, the first lane depicts the molecular weight marker with few bands marked in kDa. Panel D shows the fluorescence microscopy imaging of *M*. *smegmatis* cells expressing GFP from pMA-GFP vector. Cells were also stained with FM4-64 dye and imaged.

To assess the functionality of the three promoter systems in mycobacteria, we cloned *lacZ* reporter gene coding for β-galactosidase in pMH-His, pMA-His, and pMT-His vectors and attempted to obtain an inducible expression of the histidine-tagged β-galactosidase from these promoters in *M*. *smegmatis*. The *lacZ* gene was PCR amplified from pSD5b vector using B-gal_For and B-gal_Rev primers, and was ligated in EcoRV-linearized pMH-His plasmid, thus giving rise to pMH-βgal-His. Next, to clone *lacZ* gene under tetracycline- and acetamide-inducible promoters, we first abolished the NdeI site present inside of the cloned *lacZ* in pMH-βgal-His by site-directed mutagenesis (Fig 3A). The modified vector was then digested with NdeI and SpeI to release DNA fragment containing *lacZ* along with Kan^r^ marker (Fig 3A), which was cloned in pMT-His and pMA-His plasmids at NdeI and SpeI sites, thus giving rise to pMT-βgal-His and pMA-βgal-His, respectively (Fig 3B). All of these vectors were introduced into *M*. *smegmatis* and the expression of *lacZ* was monitored by following the protein production on a Western blot using the anti-histidine tag antibody (Fig 4). Our data show that all the promoter systems are functional. The inducible expression of *lacZ* is readily visible in the case of acetamide and tetracycline; both promoter systems offer tight regulation and show negligible expression of the target gene in the absence of inducer molecule (Fig 4A). However, with *hsp60* promoter, we see significant expression even at 37°C. Similar observation has been made before for the heat-shock promoter-mediated expression at 37°C [19]. This suggests that the promoter offers strong leaky expression even at the bacterial culture temperature. The plasmid pMH-His carrying *hsp60* promoter, earlier named as pSS1, has already been used in a previous study to complement the loss of methanol dehydrogenase gene in *M*. *smegmatis*, which is required for the bacterial growth on methanol as the sole carbon source [17]. A similar study involving complementation of a gene under *hsp60* promoter is reported elsewhere [20].

Additionally, the expression of GFP and GST tags under three given promoters was examined by Western blotting, using the anti-GFP and anti-GST antibodies, respectively (Fig 4B and 4C). The data show that GFP and GST tags readily express from these promoters, thus further confirming the applicability of these promoters in gene expression in *M*. *smegmatis*. To show the expression *in vivo*, GFP was expressed from pMA-GFP plasmid in *M*. *smegmatis* and fluorescence microscopy images were captured after 3 hours of induction. Microscopy data show that GFP is functionally active and localizes in the bacterial cytosol (Fig 4D). We wish to add that the three promoters used in the study are different from each other, and the presented data only show the functional relevance and applicability of these promoters as the mycobacterial expression systems.

## Discussion

Cloning of a gene in a desired vector and its expression are required for most studies at the cellular and molecular level. Different types of vector systems offering controlled target gene expression along with suitable reporter marker are now available for mycobacteria [9, 10, 18]. However, each of these vector systems involves a unique cloning strategy such as the necessity of specific restriction enzyme to digest both the plasmid and the target DNA, which limits their use in experiments involving gene expression in mycobacteria. Furthermore, the requirement of fusion of a reporter gene with the target makes the choice of vector even more restricted.

In this manuscript, we report the construction of nine *E*. *coli*-mycobacteria shuttle plasmids that offer the cloning and expression of target gene with three different tags (hexa-histidine, GFP, and GST) and under three different promoter systems (*hsp60*, tetracycline- and acetamide-inducible). While the two of them are inducible promoters, *hsp60* generally gives constitutive expression even at bacterial culture temperature of 37°C. Although inducible promoters have their extensive use in molecular biology, constitutive expression systems such as *hsp60* promoter also have significant applicability. Indeed, *hsp60* promoter, because of its generally constitutive expression at 37°C, has been used in several studies [21–24], suggesting strongly the utility of our designed plasmids with this promoter.

**Fig 5:**
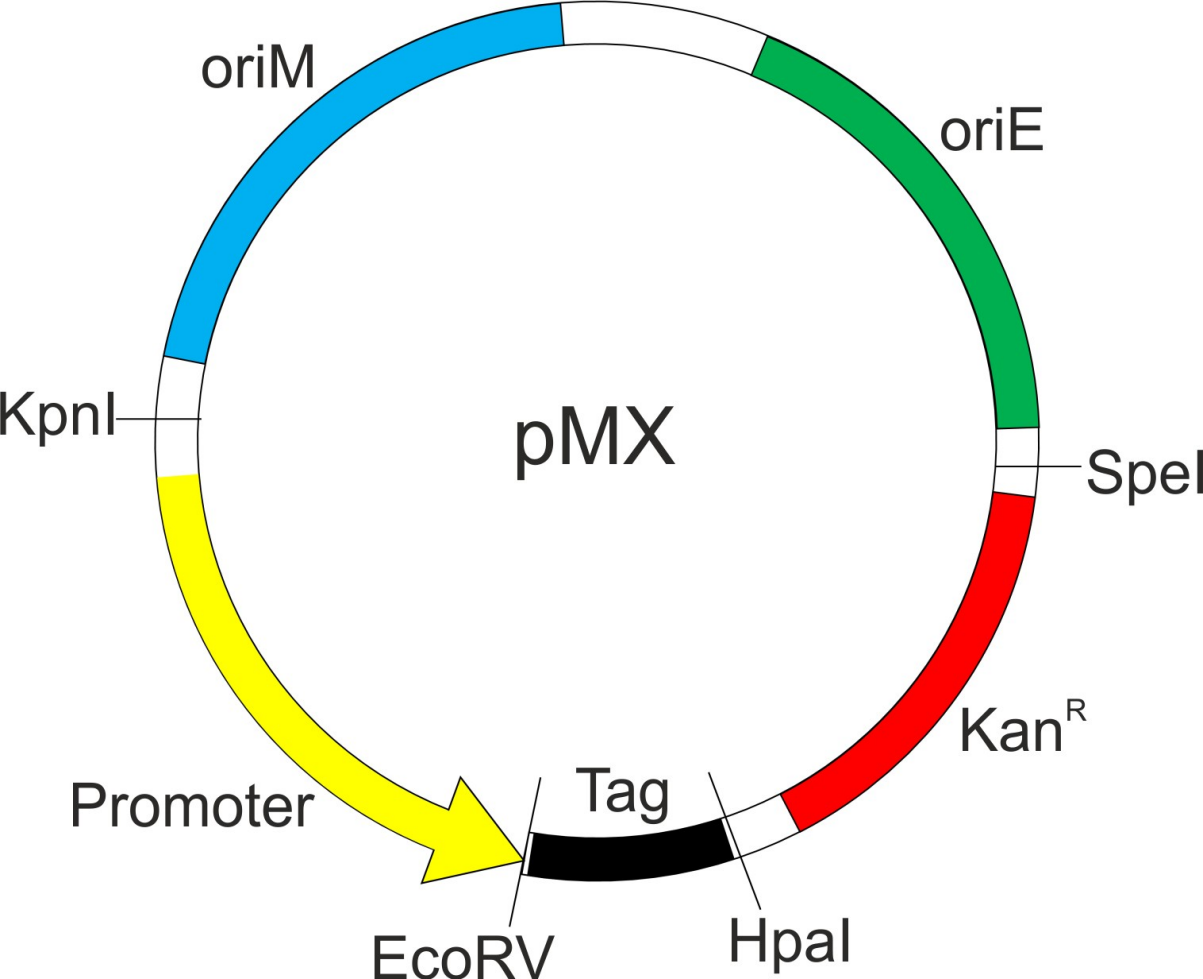
Schematic representation the proposed vector. A schematic of the vector developed in this study is shown as pMX, where X = inducible promoter system (A, acetamide; H, *hsp60*; T, *tetRO*). The plasmid retains one of the promoters (acetamidase, *hsp60*, or *tetRO*) depicted in yellow. Downstream to the promoter is the EcoRV and HpaI restriction enzyme sites that are to be used for cloning purpose. These sites sandwich one of the three tags (*viz*. 6xHis, GFP, or GST) shown in black. *oriM*, *oriE*, and Kan^R^ represent the origin of replication for mycobacteria and *E*. *coli*, and the kanamycin resistance marker, respectively.

A graphic representation of the designed plasmid is shown in Fig 5. GFP and GST are two of the most widely used tags. While the GFP is primarily used as a reporter for monitoring protein localization and live cell imaging, GST is used in the pull-down experiments for biomolecular interaction studies. The hexa-histidine tag, on the other hand, allows for the target protein purification from mycobacterial cell [25, 26], which is further useful when the target protein undergoes post-translational modification in mycobacterial cell [27]. We wish to add here that the vectors with GFP and GST tags have primarily been designed to carry out protein localization and pull-down experiments, respectively, in mycobacteria. Hence the requirement of a protease recognition site to remove the tag becomes redundant, and the same is currently not present in these vectors. The designed vectors allow for blunt-end cloning, thereby removing the requirement of a suitable restriction enzyme site for the cloning of the target DNA. Here, we have incorporated EcoRV and HpaI restriction enzyme sites in all the vectors. Digestion of a double strand DNA with these enzymes results in the digested DNA with blunt ends. Both of these enzymes site flank the tag. Therefore, to obtain the tag at the C-terminal of the target protein, cloning should be done with EcoRV enzyme, whereas HpaI enzyme should be used to install the tag at the N-terminal of the target protein. Such strategy makes the cloning simple as it eliminates the digestion of the target DNA with any enzyme.

We also wish to add that the vectors developed here offer the possibility of simultaneous cloning of the target gene in all nine vectors with the choice of either N- or C-terminal tagging. Additionally, all the tags (*viz*. 6xHis, GFP, and GST) are flanked by Ser-Gly-Ser linker, thus allowing the required flexibility between the tag and the target protein. We are now in the process of generating six more vectors that carry FLAG and RFP tags under these promoters thus yielding pMA-Flag, pMH-Flag, pMT-Flag, pMA-RFP, pMH-RFP, and pMT-RFP (data not shown) with identical features as described above. We believe that the shuttle plasmids generated here will help the scientific community in the cloning of genes and their expression in mycobacteria including the pathogenic *M*. *tuberculosis*.

## Supporting information

S1 Fig Schematic representation of the construction of pMVTet vector. Site directed mutagenesis (SDM) was carried out to introduce NdeI site in pMEND-Lx vector. The desired fragment containing *tetRO* promoter (shown in light green) was excised from the modified pMEND-Lx vector using KpnI and NdeI restriction enzymes. The released fragment was then ligated in pMV backbone prepared by digesting pMV261 vector with same enzymes.
(TIF)

S2 Fig Raw_Image. **Western blot raw images in support of Fig 4 for monitoring the expression of *lacZ* and other tags under different promoter systems.** The Western blot ram images presented here depict the production of β-galactosidase (Panel A), GFP (Panel B), and GST (Panel C). Each of these panels corresponds to the panels presented in Fig 4 as described.
(TIF)
