# Supplementary figures and images for: Construction of E. coli—Mycobacterium shuttle vectors with a variety of expression systems and polypeptide tags for gene expression in mycobacteria
Source: PLoS One. 2020 Mar 11;15(3):e0230282. doi: 10.1371/journal.pone.0230282 (PMC7065818; doi:10.1371/journal.pone.0230282)

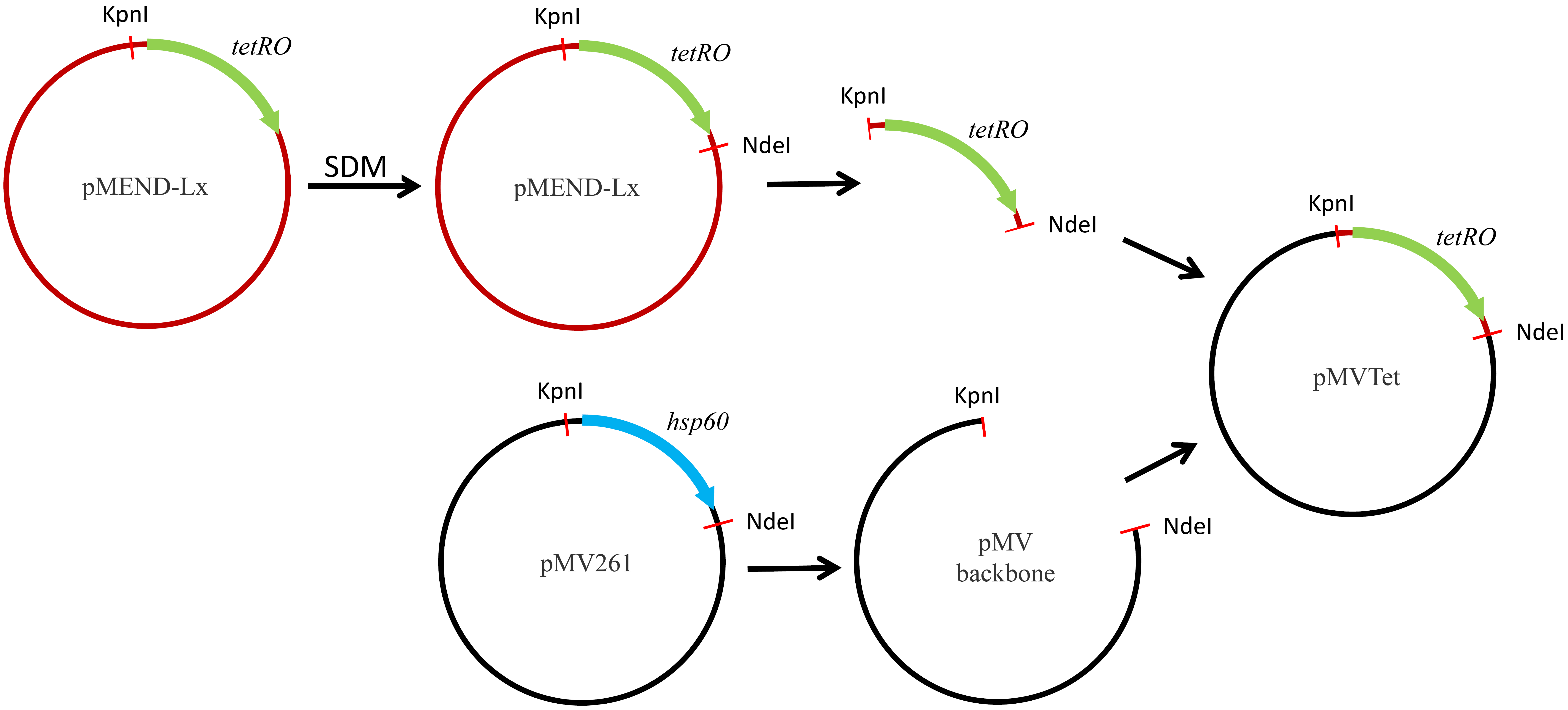

Supplement: S1 Fig — Site directed mutagenesis (SDM) was carried out to introduce NdeI site in pMEND-Lx vector. The desired fragment containing tetRO promoter (shown in light green) was excised from the modified pMEND-Lx vector using KpnI and NdeI restriction enzymes. The released fragment was then ligated in pMV backbone prepared by digesting pMV261 vector with same enzymes. (TIF) [file pone.0230282.s001.tif]

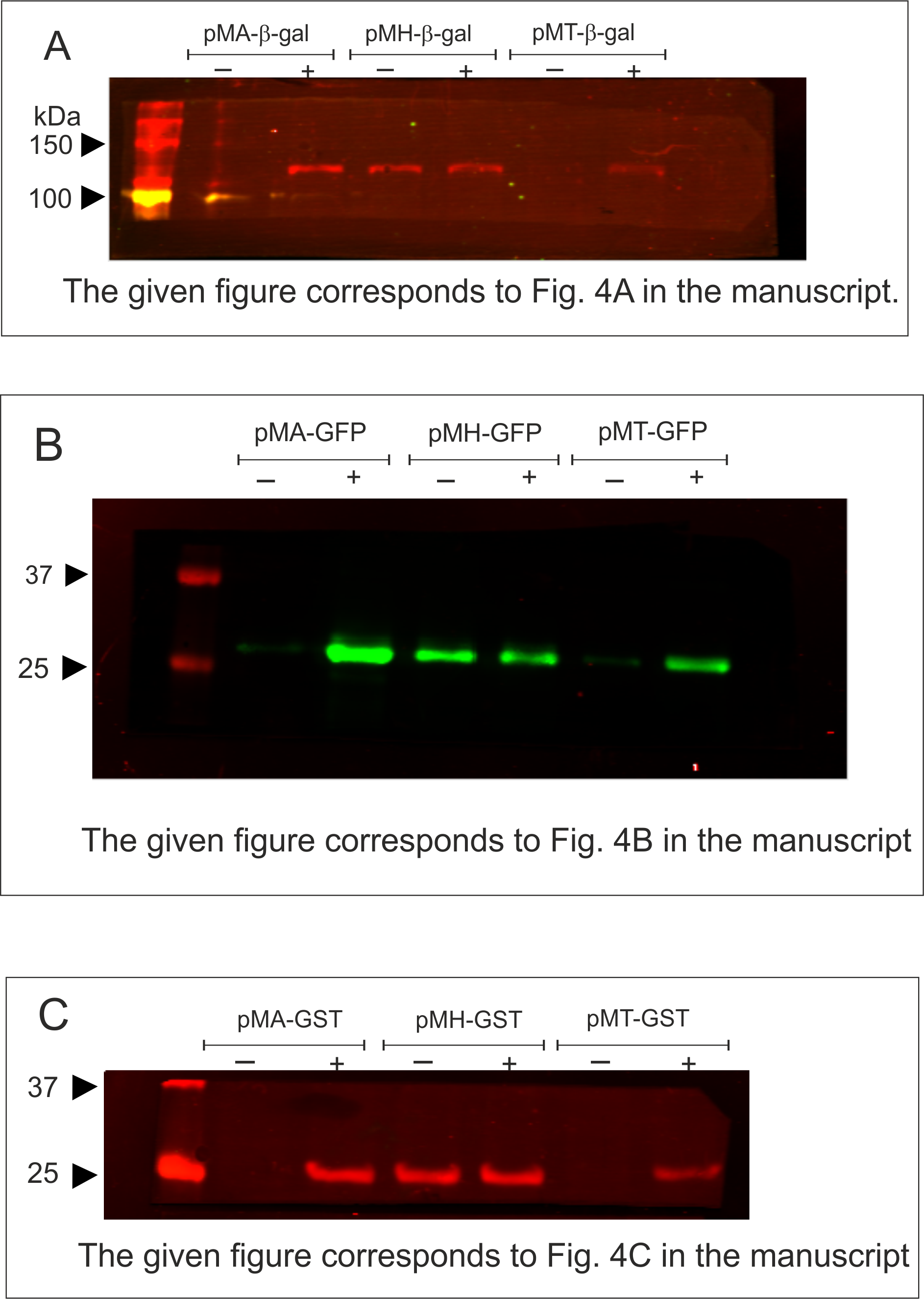

Supplement: S2 Fig — Western blot raw images in support of Fig 4 for monitoring the expression of lacZ and other tags under different promoter systems. The Western blot ram images presented here depict the production of β-galactosidase (Panel A), GFP (Panel B), and GST (Panel C). Each of these panels corresponds to the panels presented in Fig 4 as described. (TIF) [file pone.0230282.s002.tif]
